# Preclinical efficacy of the muscarinic agonist ML-007 in psychosis models depends on both M_1_ and M_4_ receptors

**DOI:** 10.1038/s41386-025-02256-3

**Published:** 2025-10-04

**Authors:** Susmita Chatterjee, Maritza Soria, Zane C. Norville, Kimberly R. Thompson, James Lillie, Anatol C. Kreitzer, Michael W. Wood

**Affiliations:** MapLight Therapeutics, Inc., Redwood City, CA USA

**Keywords:** Target validation, Pharmacology

## Abstract

Muscarinic agonists represent a new class of treatments for psychosis with a mechanism distinct from typical and atypical antipsychotics. The muscarinic subtype M_4_ has been proposed as the primary mediator of efficacy but results from recent clinical trials with M_4_-selective compounds have drawn this hypothesis into question. Instead, activation of both M_1_ and M_4_ receptor subtypes may be required for robust treatment effects. Here, we characterize the clinical-stage muscarinic agonist ML-007 in preclinical models and explore its therapeutic potential for treating psychosis in schizophrenia and Alzheimer’s disease. ML-007 is a potent brain-penetrant agonist at both M_1_ and M_4_ muscarinic receptors that has demonstrated compelling efficacy across a range of preclinical models of psychosis in schizophrenia including amphetamine-induced hyperlocomotion, PCP-induced hyperlocomotion, and conditioned avoidance response. Moreover, ML-007 is approximately ten-fold more potent than the comparator xanomeline in all animal models. Dose-response experiments in M_1_ and M_4_ knockout mice reveal that the efficacy of ML-007 is dependent on both M_1_ and M_4_ receptors. Taken together, our data suggest that both M_1_ and M_4_ receptors contribute to the potent efficacy of ML-007 in preclinical rodent models of psychosis.

## Introduction

There are five subtypes of muscarinic receptors (M_1_ -M_5_), each with distinct regional distributions and functional roles [1]. The M_1_ and M_4_ receptor subtypes show the highest expression in brain where they localize to regions implicated in psychosis such as the hippocampus, striatum, and prefrontal cortex. M_4_ receptors are linked to the regulation of locomotor activity and sensitivity to dopaminergic stimulation [2], due to expression in direct-pathway striatal projection neurons. Although robust activation of M_1_ or M_4_ alone is sufficient for efficacy in animal models of psychosis [3, 4], targeting both M_1_ and M_4_ may be more relevant for treating psychosis in schizophrenia [5]. Indeed, while M_1_ is expressed in both direct- and indirect-pathway striatal projection neurons, it appears to have greater efficacy in indirect-pathway striatal projection neurons [6] where it is co-expressed with the dopamine D_2_ receptor, the primary target of typical and atypical antipsychotics. Targeting both M_1_ and M_4_ receptors is predicted to synergistically regulate basal ganglia circuitry in a therapeutic direction more effectively than either receptor alone, and without the deleterious side effects associated with D_2_ receptor antagonism.

The cholinergic hypothesis of Alzheimer’s disease (AD) was inspired by basic scientific findings of the 1970s [7–9], and it triggered a search for new therapeutic agents aimed at modifying cholinergic signaling [10]. Clinical study results demonstrated that muscarinic agonism improved cognitive function and unexpectedly reduced behavioral symptoms of psychosis [11]. Muscarinic agonists were later reported to have activity across a range of preclinical models of psychosis [12, 13]. Clinical development, however, was impeded by peripheral side effects that led to significant tolerability issues. Two general strategies have emerged to obviate the peripheral side effects of muscarinic agonism – receptor selectivity and peripheral antagonism. It has been argued that M_2_ and M_3_ receptors are predominantly responsible for the peripheral side effects of non-selective muscarinic agonists [14], but clinical evaluation with selective muscarinic agonists suggest that this interpretation is not well supported [15]. Most prominently, the tolerability of the muscarinic agonist xanomeline has been improved by combination with a peripherally-active muscarinic antagonist, trospium in the recently approved schizophrenia treatment, Cobenfy^TM^ [16]. Clinical candidates selective for M_4_ have also been reported to treat psychosis [17]. However, results from recent clinical trials with M_4_-selective candidates have not been successful, raising questions about the potential efficacy of targeting only M_4_ [18, 19].

ML-007 is a muscarinic agonist at M_1_ and M_4_ receptors. We compared the profile of ML-007 with that of xanomeline at M_1_ and M_4_ receptors and in animal models frequently used to predict antipsychotic efficacy in schizophrenia. While ML-007 is less potent than xanomeline at M_1_ and M_4_ receptors in vitro, ML-007 is ten-fold more potent in animal models of psychosis. We conclude that ML-007 may be effective in treating psychotic symptoms in schizophrenia and AD, and that optimal efficacy for these indications may require activation of both M_1_ and M_4_.

## Materials and methods

### Drugs

ML-007 {5-((1 R,5 R)-3-azabicyclo[3.1.0]hexan-1-yl)-3-methyl-1,2,4-oxadiazole}, synthesized on demand by (Enamine, Kyiv, Ukraine), xanomeline (MedChem Express, Monmouth Junction, NJ and Tocris, Bristol, UK), d-amphetamine sulfate (Sigma Aldrich, St. Louis, MO), and phencyclidine (PCP) (Cayman Chemical Co., Ann Arbor, MI) were formulated on the day of the experiment for in vivo testing in vehicle saline (Baxter International, Deerfield, Illinois). All drugs were formulated at 10 ml/kg volume and administered acutely. Doses of 0.1, 0.3, 0.6, or 1 mg/kg of ML-007, and 1, 3, 6, or 10 mg/kg of xanomeline were administered intraperitoneally (IP). Doses of 3 mg/kg amphetamine and 5 mg/kg PCP were administered subcutaneously (SC). ML-007, amphetamine, and PCP were administered with 0-min pretreatment time and xanomeline was administered with a 10-min pretreatment time.

### in vitro assays

Aequorin-based calcium assays were conducted at EuroscreenFast [20]. Human M_1_ (NP_000729.2) and rat M_1_ (NP_542951.1) were stably expressed in CHO-K1-mt aequorin cells. Human M_4_ (NP_000732.2) and rat M_4_ (NP_113735.1) were stably expressed in CHO-K1-mt aequorin-G_α16_ cells. Briefly, stable recombinant cells grown 18 h prior to the test in media without antibiotics were detached by gentle flushing with PBS-EDTA (5 mM EDTA), recovered by centrifugation, and resuspended in assay buffer (DMEM/HAM’s F12 with HEPES + 0.1% BSA protease free). Cells were incubated at room temperature for at least 4 h with Coelenterazine h (Molecular Probes). 50 µl of cell suspension was then added to 50 µl of test compound plated in a 96-well plate. The resulting emission of light was recorded using the Hamamatsu Functional Drug Screening System 6000 (FDSS 6000). The assays were conducted with ten concentrations of test compounds, in duplicate, and in three independent experiments. Data were normalized to the responses of a maximal concentration of acetylcholine as follows: 1 µM @ hM_1_, 10 µM @ rM_1_, 100 µM @ hM_4_, and 10 µM @ rM_4_.

### Pharmacokinetic studies

PK studies were conducted at AAALAC accredited facility of Sai Life Sciences Limited, Pune, India. Satellite studies were performed at Sai (Telangana, India). All procedures of the present study were in accordance with the guidelines provided by the Committee for the Purpose of Control and Supervision of Experiments on Animals (CPCSEA) as published in The Gazette of India, December 15, 1998. Prior approval of the Institutional Animal Ethics Committee (IAEC) was obtained before initiation of the study. Briefly, healthy male C57BL/6 mice (8-10 weeks old) weighing between 25 ± 10 g were procured from Hylasco Bio-Technology (Medchal, India) and administered doses of ML-007 or xanomeline by intravenous (IV) or intraperitoneal (IP) routes. ML-007 and xanomeline and were formulated in saline. For IV doses, plasma sampling occurred at 0.08, 0.25, 0.5, 1, 2, 4, 8, and 12 h. For IP dosing, plasma, brain, and cerebrospinal fluid (CSF) sampling occurred at 0.08, 0.25, 0.5, 1, 2, 4, 8, and 12 h. Samples were stored below -70 ± 10 °C until bioanalysis. All samples were processed for analysis by protein precipitation method and analyzed with fit-for-purpose LC-MS/MS method. ML-007 LLOQ = 2.0 ng/mL for plasma, brain (ng/g), and CSF; xanomeline LLOQ = 0.5 ng/mL for plasma, brain (ng/g), and CSF. The pharmacokinetic parameters were estimated using the non-compartmental analysis tool of Phoenix® WinNonlin software (Ver 8.3, Certera, Radnor, PA). Additional details can be found in the Supplementary section along with the resulting PK time course plots.

### Behavior

#### Animals

All procedures were reviewed and approved by the Institutional Animal Care and Use Committee (IACUC) of MapLight Therapeutics. Male C57BL/6 J mice ( > 8 weeks of age) were obtained from Jackson Laboratory (Sacramento, USA) and female Tg2576 (>10 months of age): B6; SJL-Tg(APPSWE)2576Kha Rd1-tested were obtained from Taconic (New York, USA). The knockout mouse lines for Chrm1 (C57BL/6J-Chrm1^em1C^/Cya) and Chrm4 (C57BL/6J-Chrm4^em1C^/Cya) were obtained from Cyagen (China) and were bred in house using a homozygous X homozygous breeding scheme. Male knockout mice were used at >8 weeks of age. Husbandry details can be found in the Supplementary section.

#### Hyperlocomotion

The amphetamine-induced hyperlocomotion (AIH) and the PCP-induced hyperlocomotion tests were used to assess the potential antipsychotic activity of muscarinic agonists. Vehicle or treatment was administered IP immediately before SC administration of either amphetamine or PCP and mice were then placed in an open field arena (44 cm×44 cm x 20 cm) and allowed to freely explore the space while an automated video tracking system (Noldus EthoVision v17) monitored location and distance traveled for 30 min. Data are presented as the total distance traveled during minutes 5–15 after treatment. The evaluation period (5–15 min) was chosen because both drugs are rapidly cleared from plasma in mouse following IP administration (see Supplementary Fig. 3).

#### Conditioned avoidance responding

The conditioned avoidance response (CAR) test was employed to study the efficacy of antipsychotic drugs by assessing fear-based conditioned avoidance learning in rodents. Animals were trained with a conditioned stimulus (CS, light) to have an active response to avoid a negative unconditioned stimulus (US, foot shock) (Supplementary Materials). During the drug testing day, mice were weighed and administered IP with either vehicle, or treatment and then immediately placed in the active avoidance shuttle box while an automated tracking system (Maze Engineers, Cambridge, MA) monitored responses over a 50-trial session. An avoidance response was recorded if the subject left the shock chamber during the 10-second CS introduction. An escape response was recorded if the subject left the shock chamber during the 2-s US. Data are presented as the percentage of avoidance responses and as the total number of escape responses. Details on training procedure can be found in the Supplementary section.

### Statistical and data analysis

Statistical significance was tested by one-way ANOVA followed by Dunnett’s multiple comparisons test. Values for EC_50_ and E_max_ were calculated from individual experiments with technical duplicates using GraphPad Prism (La Jolla, CA). For all in vitro pharmacology data, pEC_50_ and E_max_ means and standard deviations of three independent trials were calculated. AIH ED_50_ values were calculated using baseline-correction and normalization to amphetamine-only and log transform of doses. Data were then fitted with a 4-parameter logistic equation in GraphPad Prism with top and bottom constraints (100% and 0%, respectively).

## Results

### Profiling of ML-007 and comparators at M1 and M4 receptors

ML-007 along with several comparator compounds were evaluated in an aequorin-based calcium assay format to determine the potency at M_1_ and M_4_ receptors for both human (ML-007 EC_50_ = 120 and 830 nM, respectively) and rat (EC_50_ = 340 and 1600 nM, respectively) orthologs. A general rank order in potency was observed at both subtypes and orthologs with xanomeline > oxotremorine > ML-007 > pilocarpine. All ligands had greater potency at the human ortholog than the rat ortholog at both M_1_ and M_4_ receptor subtypes (Table 1A). Relative to xanomeline, ML-007 also exhibited a slight bias towards M_1_ versus M_4_.

**Table 1:** in vitro potency of ML-007 and xanomeline in human and rat M_1_ and M_4_ aequorin assays and PK characteristics in male C57BL/6 mouse.

A					
		hM_1_	rM_1_	hM_4_	rM_4_
ML-007	pEC_50_	6.93 ± 0.04	6.47 ± 0.22	6.08 ± 0.04	5.79 ± 0.16
	E_max_ (%)	100 ± 5.2	86 ± 6.2	81 ± 8.0	66 ± 3.8
Xanomeline	pEC_50_	8.64 ± 0.16	8.31 ± 0.46	8.26 ± 0.08	8.03 ± 0.10
	E_max_ (%)	101 ± 5.6	75 ± 12	72 ± 6.6	59 ± 6.9
Oxotremorine	pEC_50_	8.07 ± 0.07	7.75 ± 0.22	7.75 ± 0.18	7.38 ± 0.17
	E_max_ (%)	92 ± 4.9	88 ± 13	89 ± 4.1	89 ± 9.1
Pilocarpine	pEC_50_	6.38 ± 0.03	6.26 ± 0.29	5.45 ± 0.07	5.59 ± 0.15
	E_max_ (%)	97 ± 4.6	91 ± 11	37 ± 2.5	24 ± 7.0

B						
	Dose (mg/kg)	Route	Plasma AUC (hr*ng/ml)	t_½_ (hours)	CSF C_max_ (ng/ml)	CSF AUC_last_ (hr*ng/ml)
ML-007	0.3	IV	100.82	0.48	nt	nt
ML-007	0.3	IP	95.34	0.46	115.26	63.51
Xanomeline	1	IV	51.25	0.18	nt	nt
Xanomeline	3	IP	13.2	0.45	BLOQ	BLOQ

(A) ML-007, xanomeline, oxotremorine, and pilocarpine were evaluated for potency against both human and rat isoforms of M_1_ and M_4_ using aequorin assays. ML-007 was seven-fold more potent at hM_1_ (120 nM) vs. hM_4_ (830 nM) and five-fold more potent at rM_1_ (340 nM) vs. rM_4_ (1.6 μM), respectively. Xanomeline was two-fold more potent at both hM_1_ (2.3 nM) vs. hM_4_ (5.5 nM) and rM_1_ (4.9 nM) vs. rM_4_ (9.3 nM). (B) Following IP dosing, plasma and CSF values were measured in samples from 3 male C57BL/6 mice for each time point (0.08, 0.25, 0.50, 1, 2, 4, 8, and 12 h). ML-007 CSF-Kp-C_max_ = 0.54; CSF-Kp-AUC_last_ = 0.67. All CSF measurements of xanomeline in CSF were below the limit of quantification (BLOQ = 0.5 ng/ml), so xanomeline CSF-Kp values were not calculable. The IV arms of the PK studies examined only plasma levels (nt = not tested). Pharmacokinetic profiles can be found in Supplementary Fig. 1.

### Pharmacokinetics of ML-007 and xanomeline in C57BL/6 mice

ML-007 and xanomeline were evaluated for pharmacokinetic properties in male C57BL/6 mice (Table 1B). Both compounds were cleared rapidly following IV dosing (i.e., t_½_ < 0.5 h), although ML-007 reached muscarinic receptors in brain at a much higher level than xanomeline. The bioavailability of the IP dose of ML-007 was 94.6% compared to 8.6% for xanomeline. The free concentration of drug in the relevant biophase (i.e., interstitial fluid of brain) was estimated using cerebrospinal fluid (CSF) sampling as previously described [21]. The ML-007 CSF concentration reached 115 ng/ml (or 698 nM) following 0.3 mg/kg IP injection, while the xanomeline CSF concentration was below the level of quantification (BLOQ = 0.5 ng/ml or <1.8 nM xanomeline) following 3 mg/kg IP injection. Thus, the molar CSF C_max_ concentration of ML-007 was more than 380 times that of the molar CSF C_max_ of xanomeline.

### Evaluation of ML-007 in animal models of psychosis: amphetamine-induced hyperlocomotion, PCP-induced hyperlocomotion, and conditioned avoidance responding

**Fig. 1:**
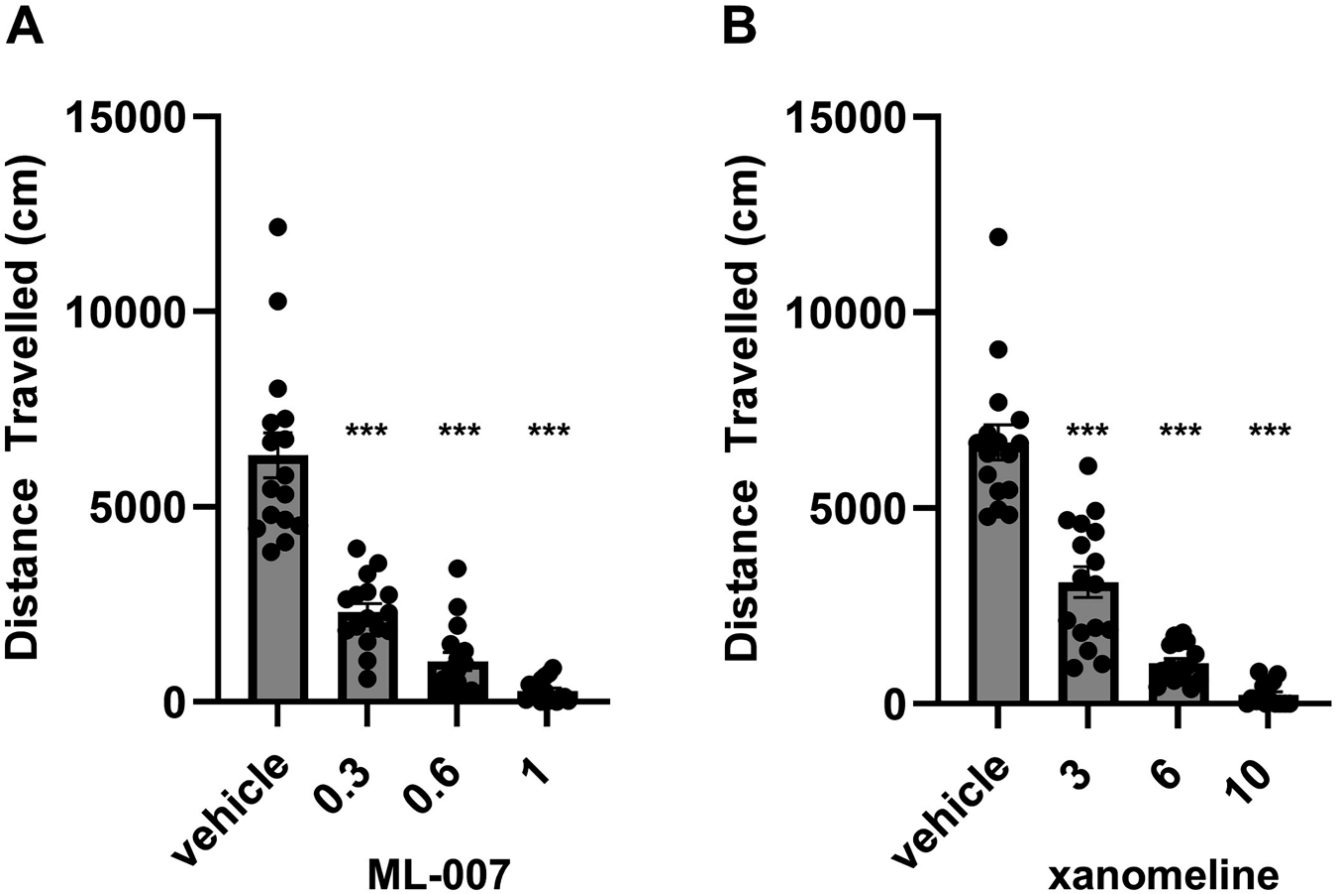
ML-007 is more potent in reversing amphetamine-induced hyperlocomotion than xanomeline. **A** Dose-dependent effects of ML-007 on reversal of AIH in mice. ML-007 (0.3, 0.6, and 1 mg/kg IP) or vehicle was administered with amphetamine (3 mg/kg IP) as two separate administrations in rapid succession and mice were immediately monitored for locomotion (n = 15–16/group, p < 0.0001). **B** Dose dependent effects of xanomeline on reversal of AIH in mice. Xanomeline (3, 6, and 10 mg/kg IP) or vehicle was administered 10 min before amphetamine (3 mg/kg IP) as two separate administrations and mice were immediately monitored for locomotion for 30 min (n = 15–16/group, p < 0.0001). Data are represented as cumulative distance over 5–15 min (*** indicates p < 0.0001 compared to vehicle-treated group, one-way ANOVA followed by Dunnett’s test, mean ± SEM).

**Fig. 2:**
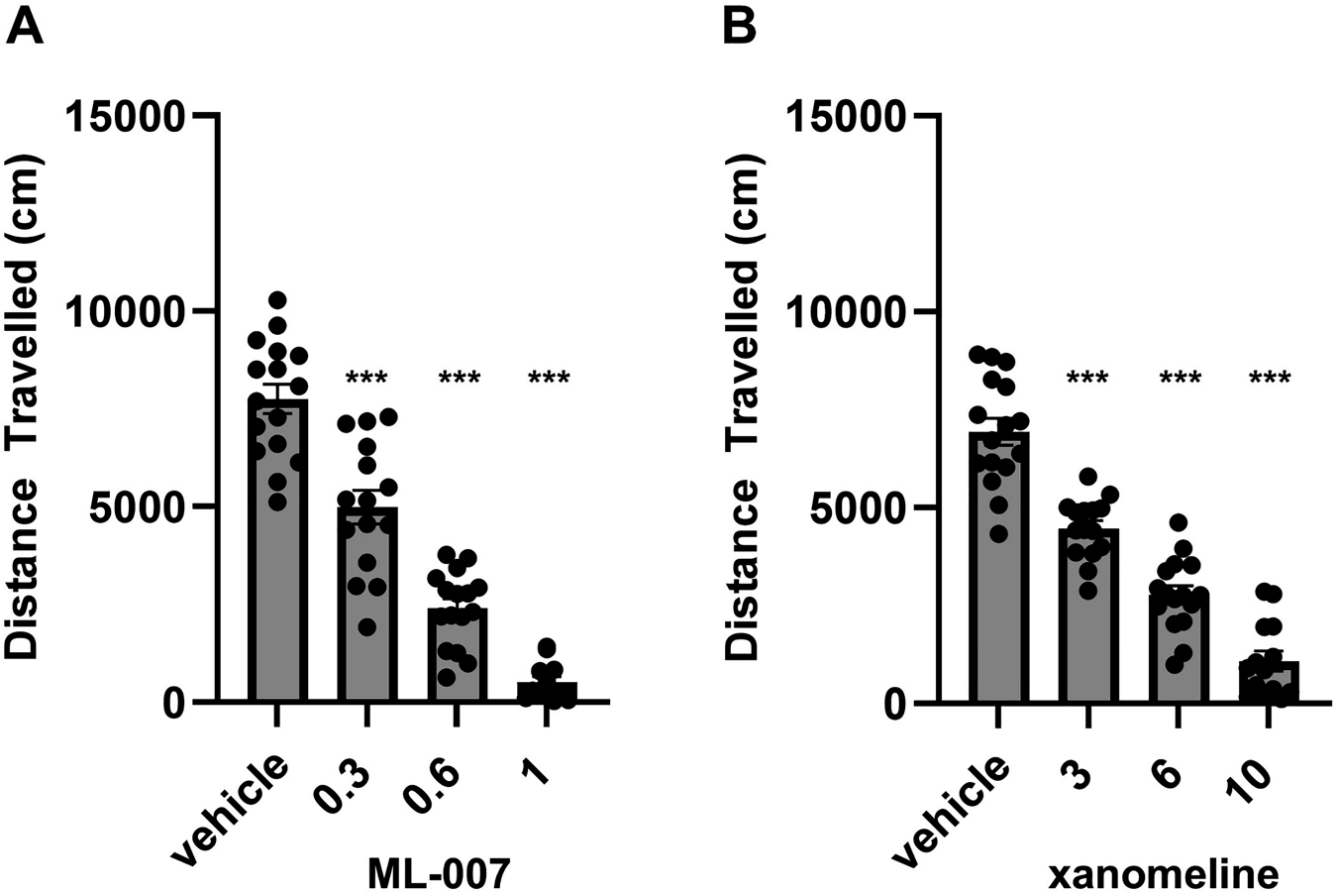
ML-007 is more potent in reversing PCP-induced hyperlocomotion than xanomeline. **A** Dose-dependent effects of ML-007 on reversal of PCP-induced hyperlocomotion in mice. ML-007 (0.3, 0.6, and 1 mg/kg IP) or vehicle was administered with PCP (5 mg/kg IP) as two separate administrations in rapid succession and monitored for locomotion for 30 min (n = 14-16/group, p < 0.0001). **B** Dose-dependent effects of xanomeline on reversal of PCP-induced hyperlocomotion in mice. Xanomeline (3, 6, and 10 mg/kg IP) or vehicle was administered with PCP (5 mg/kg IP) as two separate administrations in rapid succession and monitored for locomotion for 30 min (n = 14-16/group, p < 0.0001). Data are presented as cumulative distance over 5–15 min (*** indicates p < 0.0001 compared to vehicle-treated group, one-way ANOVA followed by Dunnett’s test, mean ± SEM).

**Fig. 3:**
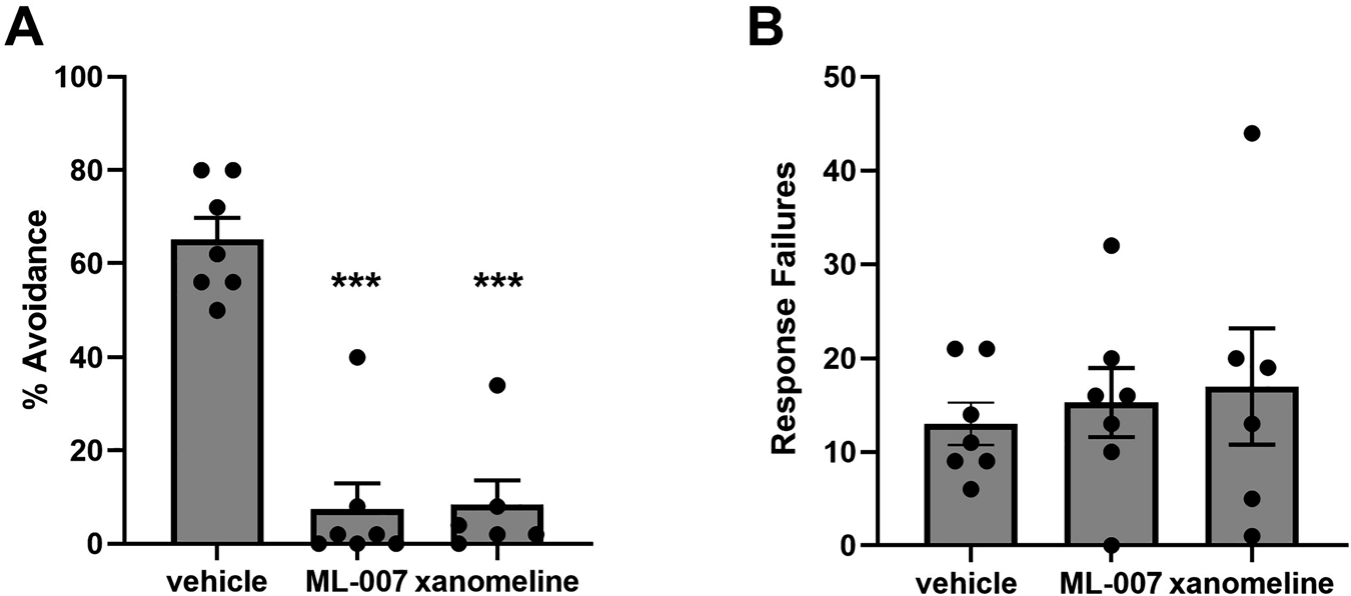
ML-007 attenuated conditioned avoidance responding at a tenfold lower dose than xanomeline. Both ML-007 and xanomeline maximally attenuated CAR without affecting the number of escape responses. 1 mg/kg ML-007 IP and 10 mg/kg xanomeline IP were administered and tested for conditioned avoidance. Data are expressed as (**A**) percent avoidance and **B** number of escape failures (n = 6–7/arm, p < 0.0001, 1-way ANOVA followed by Dunnett’s test, mean ± SEM).

To better understand the importance of the in vitro potencies and PK characteristics of ML-007 and xanomeline, we evaluated both in standard mouse models of psychosis. AIH is a precinical model that induces a transient hyperdopaminergic state, which may mimic some aspects of psychosis [22]. ML-007 exhibits a reduction of hyperlocomotion induced by amphetamine at all doses tested (0.3, 0.6, and 1 mg/kg ML-007; p < 0.001, F(3,60) = 66.22, Fig. 1A). Xanomeline showed a similar dose-dependent reduction in locomotion in the AIH assay, albeit at doses approximately tenfold higher than ML-007 (p < 0.001, F(3,59) = 85, Fig. 1B). NMDA hypofunction has also been proposed to model features of psychosis and can be induced by NMDA antagonists like PCP [23]. ML-007 showed a similar dose-dependent reduction of PCP-induced hyperlocomotion as observed in AIH (0.3, 0.6, and 1 mg/kg ML-007, p < 0.001, F(3,60) = 149.3, Fig. 2A). Xanomeline produced a dose-dependent reduction but again, across a higher dose range (p < 0.0001, F(3,57) = 87.67, Fig. 2B). The CAR assy is also commonly used to predict the antipsychotic potential of drug candidates. Approved antipsychotic drugs have been reported to block CAR without impairing escape response behavior [24]. Accordingly, we found that 1 mg/kg ML-007 inhibited avoidance responding without producing escape failures, similar to 10 mg/kg xanomeline (Fig. 3A, B, n = 6–7/arm, p < 0.001, F(2,17) = 42.22). Additionally, ML-007 was found to reverse hyperlocomotion induced by optogenetic activation of striatal direct-pathway medium spiny neurons [25], indicating that ML-007 can effectively reverse behavioral deficits resulting from altered basal ganglia circuit activity (see Supplementary Fig. 2). Overall, we observed that both ML-007 and xanomeline exhibited robust antipsychotic profiles across several experimental paradigms. Notably, the dose of ML-007 required for efficacy in animal models was approximately ten-fold lower than the dose of xanomeline required in all models.

### Contribution of muscarinic M_1_ and M_4_ activity to the effect of ML-007 on AIH

**Fig. 4:**
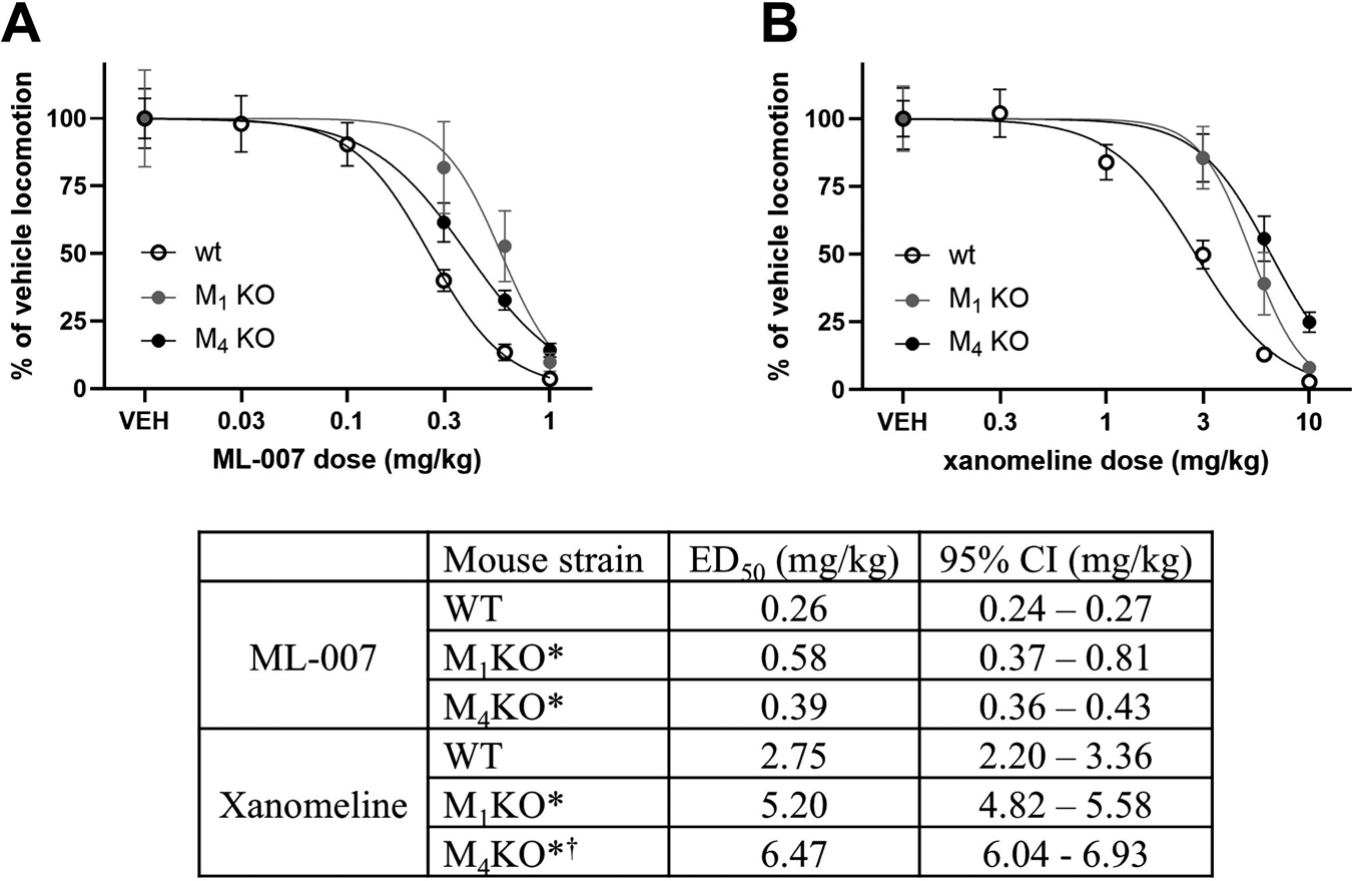
Both M_1_ and M_4_ receptors contribute to reversal of amphetamine-induced hyperlocomotion. Dose-dependent reversal of AIH normalized to vehicle with comparison across wild-type (WT), M_1_ knockout (M_1_ KO), and M_4_ knockout (M_4_ KO) mice for (**A**) ML-007 0.3, 0.6, and 1 mg/kg IP and (**B**) xanomeline 3, 6, and 10 mg/kg IP (n = 10-16/group, mean ± SEM). ED_50_ values and 95% confidence intervals (CI) were derived from fitting log-transformed data to logistics curve functions. * indicates 95% CI of compound/knockout ED_50_ does not overlap with compound/WT 95% CI. † indicates 95% CI of compound/M_4_ knockout ED_50_ does not overlap with compound/ M_1_ knockout. Raw distance traveled data can be found in Supplementary Fig. 4.

Next, we evaluated the effect of ML-007 (Fig. 4A) and the comparator xanomeline (Fig. 4B) in M_1_ and M_4_ mAChR knockout mice (M_1_ KO and M_4_ KO, respectively) and compared responses to wild-type (WT) mice to understand the relative contribution of M_1_ and M_4_ receptor activation to antipsychotic efficacy. ML-007 dose-dependently reversed AIH in both the M_1_ KO and the M_4_ KO mice. The normalized data from trials with ML-007 and xanomeline were fitted to a logistic dose-response equation (Fig. 4). For ML-007 and xanomeline, the ED_50_ in WT mice was significantly different from both the M_1_ KO and M_4_ KO mice, indicating that both M_1_ and M_4_ receptors contribute to efficacy in the AIH assay.

## Discussion

Here, we characterized the novel muscarinic agonist, ML-007, across multiple in vitro assays and in vivo rodent models relevant to psychosis. ML-007 was found to be about ten-fold more potent than xanomeline in three standard preclinical mouse models of psychosis. However, xanomeline was more potent than ML-007 at both M_1_ and M_4_ receptors in cellular aequorin assays (Table 1A). The apparent disconnect between the in vitro and in vivo potencies is a consequence of the drug properties of ML-007. ML-007 has about ten-fold greater bioavailability than xanomeline with IP administration. More importantly, dosing with ML-007 leads to a much higher free drug concentration in the brain. The ML-007 CSF C_max_ was found to be approximately 700 nM following 0.3 mg/kg IP dosing. Dosing with 3 mg/kg xanomeline produced a CSF concentration in brain that was below the limit of detection (i.e., BLOQ = 1.8 nM). CSF concentration is an accepted surrogate for free brain concentration [21] and the Free Drug Hypothesis is a well-established concept central to drug discovery and development [26]. Following IP administration of equiactive doses in mouse (0.3 mg/kg ML-007 and 3 mg/kg xanomeline), the free brain concentration of ML-007 is at least 380-fold higher than the free brain concentration of xanomeline (Table 1B). Thus, the discrepancy between the in vitro and in vivo potencies can be explained by the much higher concentration of free ML-007 in brain.

In addition to assessing ML-007 in standard animal models of psychosis, we also examined its effects in a circuit-based model of basal ganglia dysfunction that replicates some of the behavioral features of drug-induced models of psychosis. In this model, striatal direct-pathway (dopamine D_1_ receptor positive) medium spiny neurons were directly activated in vivo using optogenetics [25], which resulted in a hyperlocomotion phenotype similar to that observed with amphetamine or PCP administration. Importantly, whereas pharmacological models of psychosis engage receptors throughout the brain to induce dysfunction, optogenetics allows the experimenter to drive circuit dysfunction from a single cell type in the brain (in this case, the direct pathway basal ganglia circuit). Our results indicate that ML-007 is effective at reducing hyperlocomotion resulting from aberrant activity in basal ganglia circuitry, further localizing a functional site of action in the brain.

Profiling both ML-007 and xanomeline in M_1_ and M_4_ knockout mice revealed that high-dose activity at either M_1_ or M_4_ alone is sufficient to reverse amphetamine-induced locomotor activity. This conclusion is consistent with previous reports in which low doses of xanomeline were evaluated in M_1_ and M_4_ knockout animals [27] and in which an M_1_-selective agonist was evaluated in M_1_ knockout animals [4]. The differences in ED_50_ values between the M_1_ and M_4_ knockout mice versus the wild-type mice are modest (i.e., around two-fold for both ML-007 and xanomeline). Despite the small magnitude of shifts in ED_50_ values resulting from the absence of M_1_ or M_4_, it is evident that agonism at both M_1_ and M_4_ subtypes contributes to efficacy in preclinical models of psychosis. The hypothesis that activation of M_4_ [17] is solely responsible for the treatment effect of muscarinic agonists is not well supported by recent clinical findings [18, 19]. In contrast, the data reported here support the hypothesis that activating both M_1_ and M_4_ is more advantageous toward the treatment of psychosis than targeting either receptor alone. We have observed peripheral side effects with both ML-007 and xanomeline (e.g., salivation) and have also observed that the effects can be blocked by muscarinic antagonists (data not shown). ML-007, which activates both M_1_ and M_4_ receptors, in combination with a peripherally-acting muscarinic antagonist, is currently being evaluated in clinical development as a putative treatment for schizophrenia and psychosis in Alzheimer’s disease.

Antagonism of muscarinic receptors in the brain has long been recognized to disrupt cognitive processing [28]. The role of the M1 receptor subtype in cognition has been explored most thoroughly, and hippocampal and cortical function have been highlighted [29]. Ongoing preclinical studies are focused on understanding how ML-007 impacts cognitive processing in animal models.

## Supplementary information

Supplemenatry data

## Data Availability

Most data generated or analysed during this study are included in this published article and its supplementary information files. Datasets not included in the published article or its supplementary information files are available from the corresponding author on reasonable request.
