# Supplementary material for: Preclinical efficacy of the muscarinic agonist ML-007 in psychosis models depends on both M1 and M4 receptors
Source: Neuropsychopharmacology. 2025 Oct 4;51(5):840–5. doi: 10.1038/s41386-025-02256-3 (PMC13013942; doi:10.1038/s41386-025-02256-3)
Supplement: Supplementary file 1 — Supplemenatry data [file 41386_2025_2256_MOESM1_ESM.doc]

**Supplemental Information**

**Supplemental Methods and Materials**

**Animals in behavioral studies**

All experiments were conducted between 9:00 AM and 5:00 PM. Mice were grouped housed under a 12-h light/dark cycle (lights on at 7:00 AM), in a temperature- (20.5+2.5°C) and humidity-(55+15%) controlled environment. All experiments were done with food and water freely available. Animals were allowed to habituate to the behavior room for at least 30 minutes in the testing room. An illumination of 50 ± 20 lux was maintained during testing.

**Conditioned avoidance responding training**

Training period occurred over 4–5-days. Mice were conditioned to associate a light stimulus with foot shock to elicit an avoidance response. Mice were placed in a two-chamber shuttle box (Maze Engineers) where they were exposed to a 10-second light stimulus conditioned stimulus (CS), followed by a 2-second 0.3 mA shock, unconditioned stimulus (US) for 50 consecutive 12-second CS-US shock pairings that were separated by 30-second inter-trial intervals. The percentage avoidance was calculated for each daily session as the number of avoidance responses divided by the total number of trials (50) per day, multiplied by 100. Mice showing avoidance responses of ≥40% for two consecutive days met the criterion for successful conditioning. The total number of escape failures across the 50-trial session was also calculated. A failure response was recorded if the subject did not leave the shock chamber for the entire 12-second CS-US presentation.

**Optogenetic studies**

Stereotaxic injections of AAV5-DIO-ChR2 virus targeted the dorsal medial striatum (AP+0.8, ML+1.6, DV-3.5 from bregma, 4x10^12^ vg/ml, 600 nl/side) in 8-week-old Drd1a-Cre transgenic mice (Gensat FK150). Optical cannulas (Doric Lenses) were implanted 0.5 mm above the injection site (AP+0.8, ML+1.6, DV-3.0 from bregma), and the virus was allowed to incubate for 8 weeks prior to behavioral testing. Laser power was adjusted to yield 0.1 mW at the tip of each fiber. Vehicle or ML-007 was administered IP immediately before mice were placed in an open field arena while distance travelled was monitored by an automated video tracking system (Noldus EthoVision v17). Baseline light OFF locomotion was recorded for 5-minutes followed by 5 minutes of light ON using constant laser illumination. A crossover design was implemented in which each animal served as its own vehicle control.

**Supplemental Data**

**Supplemental Table 1. Selectivity of ML-007.**

All data from Eurofins Cerep or Eurofins Panlabs

| **Assay Target** | **Mode** | **Result Type** | **Format** | **Result** | **Site** |
| --- | --- | --- | --- | --- | --- |
| AChE | inhibitor | IC50 | Flourescent substrate | > 30 uM | Cerep |
| ADORA2A | agonist & antagonist | EC50/IC50 | Ca^2+^ | > 30 uM | Cerep |
| ADRA1A | agonist & antagonist | EC50/IC50 | Ca^2+^ | > 30 uM | Cerep |
| ADRA2A | agonist & antagonist | EC50/IC50 | cAMP | > 30 uM | Cerep |
| ADRB1 | agonist & antagonist | EC50/IC50 | cAMP | > 30 uM | Cerep |
| ADRB2 | agonist & antagonist | EC50/IC50 | cAMP | > 30 uM | Cerep |
| AR | agonist & antagonist | EC50/IC50 | PathHunter | > 30 uM | Cerep |
| AVPR1A | agonist & antagonist | EC50/IC50 | Ca^2+^ | > 30 uM | Cerep |
| CAV1.2 | inhibitor | IC50 | FLIPR Tetra | > 30 uM | Cerep |
| CCKAR | agonist & antagonist | EC50/IC50 | Ca^2+^ | > 30 uM | Cerep |
| CNR1 | agonist & antagonist | EC50/IC50 | cAMP | > 30 uM | Cerep |
| CNR2 | agonist & antagonist | EC50/IC50 | cAMP | > 30 uM | Cerep |
| COX1 | inhibitor | IC50 | Flourescent substrate | > 30 uM | Cerep |
| COX2 | inhibitor | IC50 | Flourescent substrate | > 30 uM | Cerep |
| DAT | inhibitor | IC50 | Flourescent uptake | > 30 uM | Cerep |
| DRD1 | agonist & antagonist | EC50/IC50 | cAMP | > 30 uM | Cerep |
| DRD2S | agonist & antagonist | EC50/IC50 | cAMP | > 30 uM | Cerep |
| EDNRA | agonist & antagonist | EC50/IC50 | Ca^2+^ | > 30 uM | Cerep |
| GABAA | opener & inhibitor | EC50/IC50 | FLIPR Tetra | > 30 uM | Cerep |
| GR | agonist & antagonist | EC50/IC50 | PathHunter | > 30 uM | Cerep |
| hERG | inhibitor | IC50 | FLIPR Tetra | > 30 uM | Cerep |
| HRH1 | agonist & antagonist | EC50/IC50 | Ca^2+^ | > 30 uM | Cerep |
| HRH2 | agonist & antagonist | EC50/IC50 | cAMP | > 30 uM | Cerep |
| HTR1A | agonist & antagonist | EC50/IC50 | cAMP | > 30 uM | Cerep |
| HTR1B | agonist & antagonist | EC50/IC50 | cAMP | > 30 uM | Cerep |
| HTR2A | agonist & antagonist | EC50/IC50 | Ca^2+^ | > 30 uM | Cerep |
| HTR2B | agonist & antagonist | EC50/IC50 | Ca^2+^ | > 30 uM | Cerep |
| HTR2C | agonist & antagonist | EC50/IC50 | IP1 | > 30 uM | Panlabs |
| HTR3A | agonist | EC50 | IonFlux | > 30 uM | Panlabs |
| HTR3A* | opener & inhibitor | EC50/IC50 | FLIPR Tetra | > 30 uM | Cerep |
| INSR | inhibitor | IC50 | KINOMEscan | > 30 uM | Cerep |
| KvLQT1/minK | opener & inhibitor | EC50/IC50 | FLIPR Tetra | > 30 uM | Cerep |
| LCK | inhibitor | IC50 | KINOMEscan | > 30 uM | Cerep |
| MAOA | inhibitor | IC50 | Flourescent substrate | > 30 uM | Cerep |
| nAChR (a1/b1/d/e) | agonist | EC50 | IonFlux | > 30 uM | Panlabs |
| nAChR (a3/b4) | agonist | EC50 | IonFlux | > 30 uM | Panlabs |
| nAChR (a4/b2) | agonist | EC50 | IonFlux | > 30 uM | Panlabs |
| nAChR (a4/b2)* | opener & inhibitor | EC50/IC50 | FLIPR Tetra | > 30 uM | Panlabs |
| nAChR (a7/ric3) | agonist | EC50 | IonFlux | > 30 uM | Panlabs |
| NAV1.5 | inhibitor | IC50 | FLIPR Tetra | > 30 uM | Cerep |
| NET | inhibitor | IC50 | Flourescent uptake | > 30 uM | Cerep |
| NMDAR (1A/2B) | opener & inhibitor | EC50/IC50 | FLIPR Tetra | > 30 uM | Cerep |
| OPRD1 | agonist & antagonist | EC50/IC50 | cAMP | > 30 uM | Cerep |
| OPRK1 | agonist & antagonist | EC50/IC50 | cAMP | > 30 uM | Cerep |
| OPRM1 | agonist & antagonist | EC50/IC50 | cAMP | > 30 uM | Cerep |
| PDE3A | inhibitor | IC50 | Flourescent substrate | > 30 uM | Cerep |
| PDE4D2 | inhibitor | IC50 | Flourescent substrate | > 30 uM | Cerep |
| ROCK1 | inhibitor | IC50 | KINOMEscan | > 30 uM | Cerep |
| SERT | inhibitor | IC50 | Flourescent uptake | > 30 uM | Cerep |
| VEGFR2 | inhibitor | IC50 | KINOMEscan | > 30 uM | Cerep |

**Supplemental Figure 1. Pharmacokinetics of ML-007 and xanomeline following IP administration in male C57BL/6 mice.** Pharmacokinetic profiles of ML-007 for IV & IP dosing are shown across the sampling period (i.e., 12-hours is the final time point). All CSF samples of xanomeline are below the lower limit of quantitation (LLOQ) of 0.5 ng/ml.

**A**

**B**

**Supplemental Figure 2: ML-007 reverses hyperactivity driven by optogenetic activation of striatal direct pathway medium spiny neurons.** ChR2 was expressed in direct pathway neurons via Cre-dependent AAVs injected into D1-Cre mice. (A) Locomotor activity was measured before (Light OFF) and during (Light ON) optogenetic stimulation of the direct pathway in vehicle (black) and 0.3mpk ML-007 IP treated mice (gray) (0.1mW, 5 mins constant illumination). (B) ML-007 reduced hyperlocomotion induced by optogenetic stimulation of the direct pathway (p=0.0014, paired t-test, n=19).

**A**

**B**

**Supplemental Figure 3: ML-007 and xanomeline reduce basal locomotion in a dose-dependent manner.** We compared the dose-response and time course of ML-007 and xanomeline on basal locomotion in C57BL/6 mice (n=10-15/group). ML-007 produced a dose- and time-dependent decrease in locomotion. The decreases in locomotion were found to be most robust during the first 15 minutes after administration (Fig 3A). Both ML-007 and xanomeline exhibit rapid plasma clearance in mouse following IP dosing. So, comparisons between ML-007 and xanomeline were made between 5 and 15 minutes after administration. The minimum effective dose was 0.3 mg/kg IP compared with vehicle measured between 5 and 15 minutes (Fig 3B; F [4,57] =66.73, P<0.0001). Xanomeline treatment also produced a dose-dependent decrease with a minimum effective dose at 3 mg/kg IP compared with vehicle also measured between 5 and 15 minutes (Fig. 3C, 3D; F [4,44] =26.45, P<0.0001).

**3A**

**3B**

**3C**

**3D**

**Supplemental Figure 4: Raw data from Figure 4. Both M1 and M4 receptors contribute to reversal of amphetamine-induced hyperlocomotion.** ML-007 (0.3-1 mg/kg IP, 4A & 4C) or xanomeline (3-10 mg/kg IP, 4B & 4D) were administered with amphetamine (3 mg/kg IP) as two separate administrations in rapid succession and immediately monitored for locomotion for 30 mins in M1KO (A&B) and M4KO (C&D). Data are presented as cumulative distance over 5 to 15 min (n=10-16/group). Distance traveled and corresponding p-values can be found in Supplemental Table 2.

**4A**

**4B**

**4C**

**4D**
